# Epigenetic response of imprinted domains during carcinogenesis

**DOI:** 10.1186/s13148-017-0393-8

**Published:** 2017-08-25

**Authors:** Corey L. Bretz, Ingeborg M. Langohr, Joomyeong Kim

**Affiliations:** 1 Department of Biological Sciences, Louisiana State University, Baton Rouge, LA 70803 USA; 2 Department of Pathobiological Sciences, Louisiana State University School of Veterinary Medicine, Baton Rouge, LA 70803 USA

**Keywords:** DNA methylation, Genomic imprinting, Imprinting control regions, Imprinted genes, Cancer, Carcinogenesis, Squamous papilloma, T cell lymphoma

## Abstract

**Background:**

Imprinted domains have been identified as targets for aberrant DNA methylation during carcinogenesis, but it remains unclear when these epigenetic alterations occur and how they contribute to tumor progression. Epigenetic instability at key *cis-*regulatory elements within imprinted domains can concomitantly activate proto-oncogenes and turn off tumor suppressor genes. Thus, to further characterize the epigenetic response of imprinted domains during carcinogenesis, we compared the stability of DNA methylation at a variety of *cis*-regulatory elements within imprinted domains in two fundamentally different mouse tumors, benign and malignant, induced by the *KrasG12D* mutation.

**Results:**

We report that imprinted domains remain stable in benign processes but are highly susceptible to epigenetic alterations in infiltrative lesions. The preservation of DNA methylation within imprinted domains in benign tumors throughout their duration suggests that imprinted genes are not involved with the initiation of carcinogenesis or the growth of tumors. However, the frequent detection of DNA methylation changes at imprinting control regions in infiltrative lesions suggest that imprinted genes are associated with tumor cells gaining the ability to defy tissue boundaries.

**Conclusion:**

Overall, this study demonstrates that imprinted domains are targeted for DNA hypermethylation when benign tumor cells transition to malignant. Thus, monitoring DNA methylation within imprinted domains may be useful in evaluating the progression of neoplasms.

**Electronic supplementary material:**

The online version of this article (doi:10.1186/s13148-017-0393-8) contains supplementary material, which is available to authorized users.

## Background

1Imprinted genes are mainly expressed from a single allele based upon the allele’s parental origin [1]. Thus far, there have been just over 100 imprinted genes identified by conventional methodology; however, a far greater number of imprinted genes may exist due to recent data obtained by implementing modern whole-transcriptome sequencing technologies [2, 3]. Nevertheless, the well-known imprinted genes are clustered in discrete chromosomal domains, which often contain a differentially methylated master *cis*-regulatory element termed an imprinting control region (ICR) [1, 4, 5]. ICRs are accompanied by other *cis*-regulatory elements such as enhancers and differentially methylated promoters that act synergistically within their respective imprinted domain to elegantly control imprinted gene dosage [1, 6]. While DNA methylation is transient among the other *cis*-regulatory elements depending on developmental contexts and cell types, DNA methylation at ICRs is exceedingly stable [1, 6]. The stability of this epigenetic mark at ICRs is paramount due to their control over several imprinted genes within a domain that have key roles in cell growth, division, or death [7–9]. Consistent with this, imprinted genes have gained much attention as both tumor suppressor genes and oncogenes [10–14]. Indeed, epigenetic perturbations within imprinted domains have been reported in a variety of both human and mouse malignant neoplasms [15, 16]. However, the timing in which epigenetic change occurs within imprinted domains, especially at ICRs, is still unclear.

DNA methylation is an important epigenetic regulator of gene expression. Misregulation of DNA methylation in tumor cells is well recognized as an epigenetic alteration that results in significant expression level differences of genes that contribute to the carcinogenic process [13, 14, 17–20]. The genome of any given normal cell by default is virtually stifled with DNA methylation, with the exception of specific gene promoters and *cis*-regulatory elements such as enhancers that contribute to the expression of genes necessary for the proper function of the respective cell. However, during carcinogenesis, the levels of DNA methylation are drastically reduced genome wide [21]. There are two distinct drastic reductions in 5-methylcytosine that occur throughout the genome during the carcinogenic stages: first, during the transition of normal cells to immortalized tumor cells, and second, as benign tumor cells acquire infiltrative capacity [21]. Interestingly, hypermethylation at specific regulatory elements such as tumor suppressor gene promoters occurs concomitantly with genome-wide hypomethylation during these critical transitional stages. Although ICRs have been well identified as targets for hypermethylation, the functional role that this epigenetic aberration plays during the carcinogenesis process remains largely uncharacterized [15, 16]. Does hypermethylation at ICRs contribute to the initiation of tumor formation, the proliferation of tumor cells, or the transition of a benign process to that of an infiltrative one?

In the current study, we exploited the *KrasG12D* genomic mutation to initiate carcinogenesis in mice and surveyed the epigenetic stability at ICRs in two fundamentally different neoplasms: squamous papilloma that remains a benign process but exhibits continual growth, and T cell lymphoblastic lymphoma that rapidly acquires infiltrative capacity. This comparison allowed further characterization of the functional role that aberrant DNA methylation at ICRs plays during the carcinogenic process. According to the results, ICRs remain epigenetically stable during the initiation of carcinogenesis and throughout the growth of tumors. However, DNA hypermethylation was observed across virtually all ICRs in infiltrative tumor cell populations regardless of their duration. These data suggest ICRs are targeted during the second phase of DNA methylation alterations to the genome and epigenetic instability among ICRs contributes to the infiltrative capacity of tumor cells. Detailed results and the relevant discussion are presented below.

## Results

**Fig. 1:**
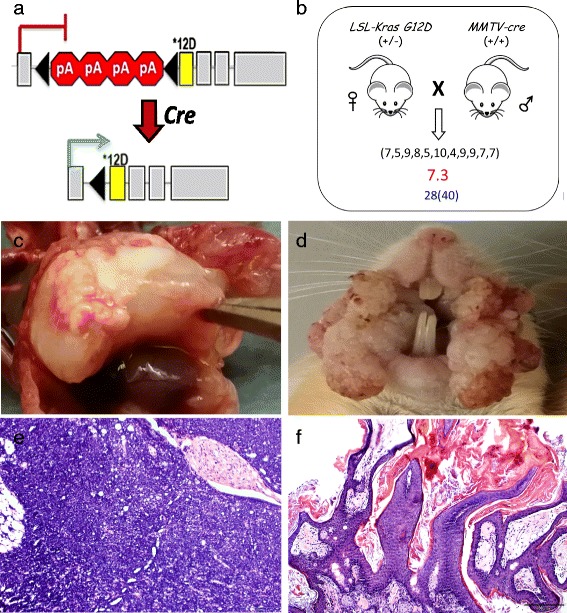
Targeting oncogenic *KrasG12D* to mouse T cells and epithelial cells. **a** Structure of the conditional *LSL-Kras* ^*G12D*^ allele. Once Cre removes the transcriptional stop cassette, transcription of oncogenic *KrasG12D* proceeds. **b** Breeding scheme involving mouse lines *LSL-Kras* ^*G12D*^ and MMTV-Cre. The numbers in parentheses indicate how many pups were born in each litter, the red number indicates the average litter size, and the blue numbers indicate how many mutant mice were obtained out of the expected number based on Mendelian inheritance. **c** Gross image of a thymic T cell lymphoma that is markedly expanding the anterior mediastinum in a KrasG12D mouse. **d** Gross image of a squamous papilloma in and around the mouth of a KrasG12D mouse. **e** Histological image of a thymic T cell lymphoma in a KrasG12D mouse with neoplastic lymphoid cells completely effacing the normal architecture of the thymus and surrounding mediastinal soft tissue (200× magnification). **f** Histological image of a squamous papilloma in a KrasG12D mouse showing the proliferated epithelium forming multiple finger-like projections with maintenance of the tissue boundaries (100× magnification)

In this study, we sought to test whether the instability of DNA methylation at imprinting control regions (ICRs) is primarily associated with the transition of tumor cells to more infiltrative states. We also sought to compare the relative stability of DNA methylation at ICRs with that of other regulatory sequences such as evolutionarily conserved regions (ECRs) that are known to be putative enhancers and associated with the ICR of their respective imprinted domain. To accomplish this, we utilized a floxed allele that contains the oncogenic *KrasG12D* mutation to initiate tumorigenesis. The *LSL-Kras* ^*G12D*^ allele contains a Lox-Stop-Lox (LSL) transcriptional stop cassette within the first intron, and a point mutation in the second exon that causes a substitution (glycine to aspartic acid) at the 12th amino acid of the *KRAS* protein (Fig. 1a) [22, 23]. Upon the activity of cyclic recombinase (Cre), the stop cassette is removed from the first intron allowing transcription to proceed and produce a full-length transcript that encodes the oncogenic *KRASG12D* protein.

The *KrasG12D* mutation is sufficient to initiate both an infiltrative T cell neoplasm and benign squamous cell papilloma [15, 24, 25]. Furthermore, the KrasG12D mutation has also been exploited to study non-small cell lung cancer, pancreatic cancer, and colorectal tumors. Previously, we reported the epigenetic instability at ICRs within T cell lymphoma driven by the *KrasG12D* mutation, which suggested that ICRs are targeted for hypermethylation in infiltrative T cell populations [15]. With the current study, we sought to further support the previous report by comparing the T cell neoplasm to a benign or non-infiltrative tumor type. To accomplish this, we bred mice that express Cre under the Mouse Mammary Tumor Virus long terminal repeat promoter (MMTV-Cre) with the *LSL-Kras* ^*G12D*^ line to simultaneously target oncogenic *KrasG12D* to T cells and epithelial cells of the mucous membranes (Fig. 1b) [26, 27]. Using this breeding scheme, we obtained 80 mice from 11 litters with an average litter size of 7. Of the 80 total mice, 28 were positive for the recombined *KrasG12D* allele, which deviated significantly from the expected Mendelian ratio (*P* = 0.007, chi-square). All of the recombinant progeny developed squamous papilloma and nearly 40% developed thymic lymphoma as well as squamous papilloma (Fig. 1c, d) [15]. Unlike the thymic lymphoma, which infiltrated and extensively replaced the surrounding tissues (Fig. 1e), the squamous papillomas were benign. The squamous papilloma lesions retained an exophytic growth pattern with an overall thickening of the epithelial layer into finger-like projections (Fig. 1f). In sum, a mouse breeding scheme utilizing *KrasG12D*/*MMTV-Cre* mice was successful in obtaining mice that developed both benign and infiltrative lesions.

**Fig. 2:**
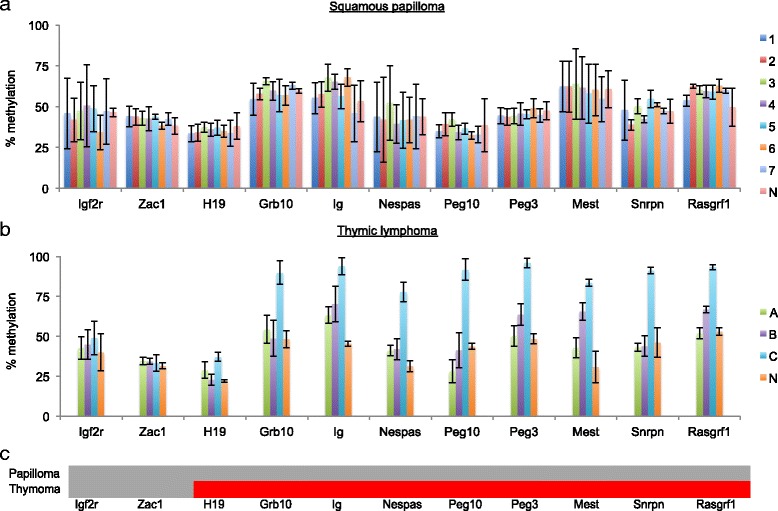
DNA methylation signatures at ICRs in benign squamous papilloma and infiltrative thymic T cell lymphoma. **a** Mean percent methylation with 95% confidence intervals from 11 ICRs in squamous papilloma generated by COBRA. Data from 7 out of the 15 squamous papilloma tumors are shown and compared to a normal sample denoted with an N. **b** Mean percent methylation with 95% confidence intervals from 11 ICRs in thymic lymphoma generated by COBRA. Data from 3 representative thymic lymphoma samples (A—hyperplastic, B—atypical hyperplastic, and C—neoplastic) are compared to normal thymus tissues labeled N. **c** Summary of DNA methylation changes at the 11 ICRs in papilloma and thymoma. Red represents significant hypermethylation based on the samples having a *P* value less than 0.05, and gray represents no significant DNA methylation changes detected

**Fig. 3:**
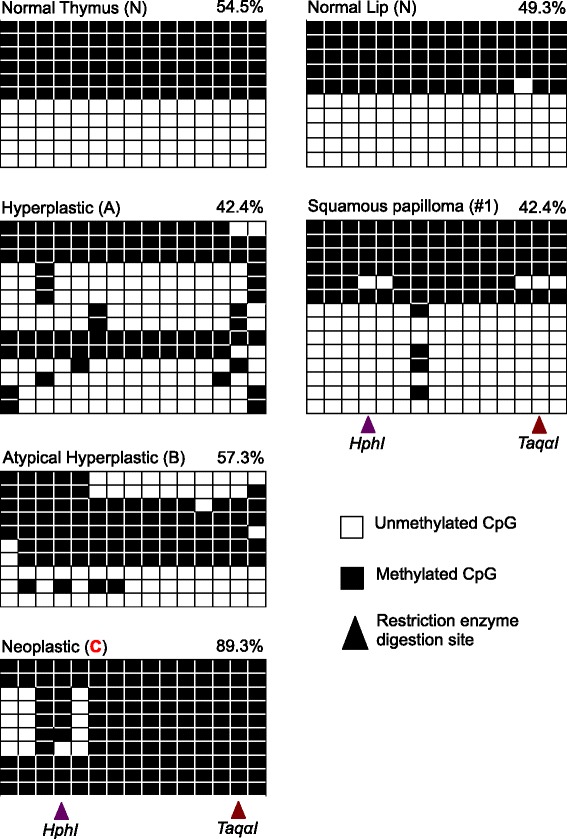
Bisulfite sequencing of the Peg3 imprinting control region. Each row represents an individual sequencing result from a clone. Each column represents a CpG site within the Peg3-ICR PCR product. The black boxes denote methylated CpG cites. The white boxes denote unmethylated CpG sites. The restriction enzyme digestion sites within the PCR products are marked with triangles: purple for *Hph*I and red for *Taqα*I. Red coloration of the sample name/number indicates significant methylation changes detected by both COBRA and sequencing

DNA methylation at ICRs remained stable with the 50% methylation levels in benign tumor cells, but became unstable with hypermethylation showing greater than 50% methylation levels in infiltrative T cell tumor cells (Fig. 2a, b). We sampled 15 squamous papilloma tumors (7 of which from varying duration were selected for statistical analyses) and measured the DNA methylation levels by COmbined Bisulfite Restriction Analysis COBRA at 11 ICRs, including *Igf2r*, *Zac1*, *H19*, *Grb10*, *Ig*, *Nespas*, *Peg10*, *Peg3*, *Mest*, *Snrpn*, and *Rasgrf1* [15, 28–30]. The 15 tumors varied in duration from 1 to 4 months and thus size from small to large. Nevertheless, DNA methylation at ICRs remained stable throughout the growth of all the squamous papilloma tumors. This was apparent, as the ratio of digested bisulfite PCR amplicons (representing methylated DNA) to undigested amplicons (representing unmethylated DNA) in the tumor samples was not significantly different from the ratio of normal tissue samples (Fig. 2a, Additional file 1: Figure S1). We then compared these methylation data in squamous papilloma to that of thymic lymphoid proliferative lesions histologically determined to be hyperplastic, atypical hyperplastic, and neoplastic [15, 31, 32]. While DNA methylation at ICRs remained stable in hyperplastic and atypical hyperplastic cells, it became unstable in infiltrative neoplastic cells (Fig. 2b, c, Additional file 1: Figure S1). COBRA detected significant DNA hypermethylation at 9 of the 11 ICRs tested in the infiltrative T cell samples (*C*) compared to normal thymus samples (*N*), including *H19* (*C* − *N* = 15%), *Grb10* (*C* − *N* = 41.6%), *Ig* (*C* − *N* = 50.2%), *Nespas* (*C* − *N* = 46.5%), *Peg10* (*C* − *N* = 47.9%), *Peg3* (*C* − *N* = 47.6%), *Mest* (*C* − *N* = 52.7%), *Snrpn* (*C* − *N* = 44.9%), and *Rasgrf1* (*C* − *N* = 40.2%) (Fig. 2b, c). Mean percent methylation values with 95% confidence intervals for each sample and locus are summarized in Additional file 2: Table S1. To validate the results from COBRA, we performed bisulfite sequencing of the Peg3-ICR (Fig. 3). The sequencing results were in agreement with those from COBRA with respect to overall DNA methylation levels. However, the sequencing profiles do reveal a mosaic pattern of DNA methylation among the CpG sites in the tumor samples compared to the normal sample that showed a clear division between methylated strands and unmethylated strands. In sum, the stability of DNA methylation at ICRs displayed a major difference between tumor cells of different states, benign versus infiltrative.

**Fig. 4:**
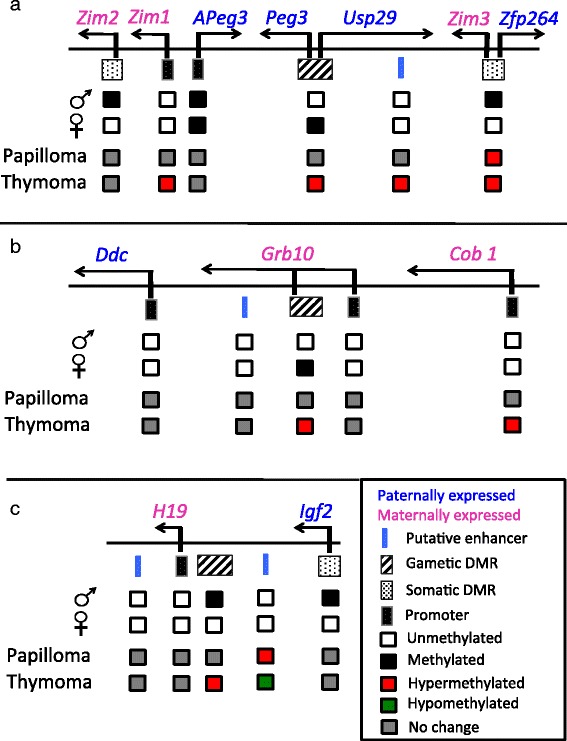
DNA methylation signatures of various sequence elements within imprinted domains in squamous papilloma and thymoma. **a** *Peg3* domain structure. Sequence elements analyzed include *Zim2*—differentially methylated region (DMR), *Zim1* promoter, *APeg3* promoter, *Peg3*—imprinting control region (ICR), ECR18, and *Zim3*/*Zfp264*—DMR. **b** *Grb10* domain structure. Sequence elements analyzed include *Ddc*—promoter, ECR154, *Grb10*—ICR, *Grb10* promoter, and *Cob1* promoter. **c** *H19*/*Igf2* domain structure. Sequence elements analyzed include ECR2, *H19* promoter, *H19*—ICR, ECR1, and *Igf2*—DMR. All sequence elements are listed in order as they appear in the figure from left to right

Certain regulatory sequence elements within imprinted domains became epigenetically unstable in the nonmalignant setting. We surveyed the DNA methylation status of various regulatory sequence elements (ICRs, somatic differentially methylated regions (DMRs), ECRs, and promoters) within three imprinted domains (*Peg3*, *Grb10*, and *H19*) to compare the epigenetic stability of the ICRs to other sequence elements within their respective imprinted domains (Fig. 4a–c). We found that there was no clear trend as to which type of sequence element first experiences epigenetic change; rather, certain sequence elements experienced significant changes in DNA methylation in the benign squamous papilloma lesions. Specifically, the *Zim3*/Zfp264 promoter of the *Peg3* domain became significantly hypermethylated in 5 out of 15 tumors (Additional file 3: Figure S2, Additional file 4: Figure S3), whereas the ICR and ECR18 of the *Peg3* domain showed no significant change in DNA methylation (Fig. 4a, Additional file 4: Figure S3). On the contrary, the ECR1 of the *H19*/*Igf2* domain became significantly hypermethylated in 2 out of 15 tumors (Additional file 3: Figure S2, Additional file 4: Figure S3), whereas none of the other sequence elements in the domain showed significant change in DNA methylation (Fig. 4c, Additional file 4: Figure S3). While certain sequence elements in both the *Peg3* and *H19*/*Igf2* domains experienced significant changes in DNA methylation, all sequence elements in the *Grb10* domain remains stable in all squamous papilloma lesions (Fig. 4b, Additional file 4: Figure S3). Mean percent methylation values with 95% CIs for each locus are summarized in Additional file 4: Figure S3. In sum, the DNA methylation at all four elements tested, ICRs, DMRs, promoters, and ECRs, behave similarly during carcinogenesis, although some elements are affected earlier than others in individual cases.

## Discussion

Many efforts have been focused on determining the potential role that imprinted genes may play during carcinogenesis [10, 11, 15, 16, 21, 33, 34]. Analyses of human malignant neoplasms have revealed that many ICRs and putative enhancers within imprinted domains show significant epigenetic change pertaining to DNA methylation and that these changes often correlate with the expression level differences of nearby imprinted genes within the domain [16]. However, the question whether this epigenetic instability within imprinted domains contributes to the infiltration potential of tumor cells remains elusive [15]. In the current study, we initiated tumorigenesis in mice with the *KrasG12D* mutation and subsequently compared the epigenetic stability of various regulatory sequence elements within imprinted domains between two fundamentally different neoplasms, benign squamous papilloma, and malignant T cell lymphoma. Although there is some overlap with our previous report on the T cell lymphoma, we further show with this report that there is a stark contrast in the epigenetic stability at ICRs between the benign and infiltrative lesions studied: while DNA methylation among ICRs was stable in the benign lesions, including in the thymic lymphoid hyperplastic lesions, it was highly unstable in the infiltrative thymic T cell lesions. The epigenetic instability at imprinting control regions was also seen in our previous report on the infiltrative thymic lymphoma, which were isolated from mice using a different breeding scheme involving a conditional knockout construct for *Peg3* that was bred into the *KrasG12D* model. Thus, it should be noted that even though the tumors from each study were isolated from mice of two different breeding schemes, the thymic tumors in both studies were of the same genotype (*MMTV-Cre*/*KrasG12D*). We also expanded our analysis of imprinted domains in the current study and show that DNA methylation is unstable at certain regulatory sequence elements specific to their imprinted domain in both the benign and malignant lesions. Overall, it appears that imprinted domains are most vulnerable to epigenetic change in infiltrative tumor cells.

DNA methylation settings at ICRs are critical for maintaining parent-of-origin specific expression of many genes within an imprinted domain [4]. ICRs contain various sequence elements that commonly manifest aberrant DNA methylation trends in tumor cells [18]; thus, the stability of DNA methylation at ICRs is concerning in the context of carcinogenesis. Moreover, ICRs are susceptible to the Knudson two-hit hypothesis because one allele is already methylated and silenced in the native state. Therefore, ICRs can potentially respond to either hypomethylation events or hypermethylation events. Indeed, the Knudson two-hit hypothesis has been well supported with hypermethylation of imprinted genes in malignant tumors [13, 15, 16]. However, the timing when ICRs manifest epigenetic change, particularly DNA methylation changes, is debatable. First, do epigenetic changes at ICRs coincide with the initiation of carcinogenesis or, second, do epigenetic changes at ICRs coincide with neoplastic transformation of tumor cells?

**Fig. 5:**
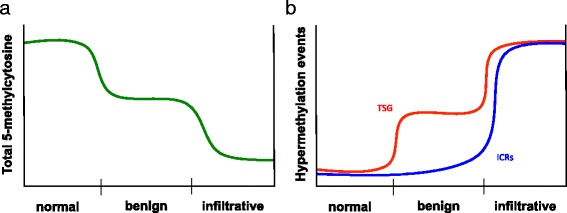
DNA methylation trends during carcinogenesis. **a** The genome-wide reductions in 5-methylcytosine during the transition of normal cells to benign tumor cells (first hash mark) and when benign cells gain infiltrative potential (second hash mark). **b** Specific hypermethylation events that coincide with the genome-wide hypomethylation trends. The red line represents hypermethylation at tumor suppressor gene (TSG) promoters and the blue line represents hypermethylation at imprinting control regions (ICRs)

According to the results of our analyses of the squamous papillomas, DNA methylation at ICRs remained stable throughout the duration of these benign tumors (Fig. 2). This suggests that epigenetic instability at ICRs is not associated with the early events of carcinogenesis. However, this epigenetic stability is particularly intriguing given that two genome-wide DNA demethylation events take place in mouse skin carcinogenesis: first, during transition from immortalized non-tumorigenic keratinocytes to benign papilloma cells and, second, during transition from epithelial to spindle cells, which is associated with a sharp increase in infiltrative potential (Fig. 5) [21]. Moreover, these genome-wide reductions in 5-methylcytosine are accompanied by two waves of DNA hypermethylation at specific tumor suppressor gene promoters [21]. Unfortunately, we were unable to analyze the epigenetic activity at ICRs during the second wave of DNA methylation changes in the squamous papilloma panel as these epithelial cells did not undergo the epithelial-mesenchymal transition. Furthermore, these squamous tumors did not even advance to squamous cell carcinomas. Nevertheless, the epigenetic stability at ICRs throughout the first reconstruction of the epigenome suggests imprinted genes do not have a role in the initiation of carcinogenesis.

Epigenetic stability at ICRs is challenged during the second reconstruction of the DNA methylome (Fig. 5). This wave of DNA methylation changes is associated with increase in the infiltrative potential of tumor cells. This is demonstrated by the results from the T cell lymphoblastic lymphoma panel (Fig. 2). In addition to neoplastic thymic lymphoid lesions, this panel also included hyperplastic and preneoplastic thymic lymphoid lesions [15]. ICRs remained epigenetically stable in the hyperplastic and preneoplastic specimens regardless of the duration of the lesions. In contrast, malignant neoplastic specimens displayed marked epigenetic instability with nearly all the ICRs becoming hypermethylated, regardless of the duration of the lesion. ICRs acquiring hypermethylation do not necessarily confer repression of imprinted genes as seen with hypermethylation in tumor suppressor gene promoters [33]. For instance, hypermethylation at the ICR of the *H19*/*Igf2* domain can potentially result in complete repression of *H19* and bi-allelic expression of *Igf2*, a known oncogene, whereas hypermethylation at the ICR of the *Peg*3 domain can potentially result in repression of *Peg3*, a putative tumor suppressor [5]. Thus, concomitant hits to multiple ICRs can simultaneously activate oncogenes while suppressing tumor suppressor genes. Overall, it appears ICRs are targeted to be hypermethylated in the midst of the second drastic genome-wide reduction in 5-methylcytosine, a hallmark of tumor progression to malignant disease. This raises the notion that epigenetic instability among ICRs could enhance the infiltrative capacity of tumor cells.

The epigenetic stability among the ICRs in the benign tumors is not a common theme among other regulatory sequences within their respective imprinted domain. This is demonstrated by our analysis of the *H19*/*Igf2* and *Peg3* imprinted domains (Fig. 4). For instance, the *Zfp264* promoter within the *Peg3* imprinted domain became hypermethylated in several benign specimens while the ICR retained normal DNA methylation patterns. This observation demonstrates the specificity of DNA methylation changes among sequence elements within imprinted domains during tumor progression. The observed specificity of DNA methylation changes within a single imprinted domain further supports the notion that epigenetic changes at ICRs could potentially be involved in enhancing the infiltrative potential of tumor cells as ICRs are only targeted during the second reconstruction of the DNA methylome.

There are limitations to consider with the methodology used to measure DNA methylation for the current study. First, COBRA is not a very sensitive method and it only measures the methylation at one CpG site at a time. Although densitometry can be performed to quantify COBRA data as we did in the current study, COBRA is also inherently qualitative. To address these issues and to validate the results from COBRA, we performed bisulfite sequencing of the *Peg3* bisulfite PCR products. The results from sequencing reinforced the results from COBRA; however, there are also limitations to consider with traditional bisulfite sequencing due to the low number of reads compared to Mass Array or Next Generation sequencing approaches. Again, caution should be used when interpreting the results from bisulfite sequencing, as they are more qualitative than quantitative. For studies that require more quantitative methodologies such as in the case with human cancer studies, it is advised to employ more sensitive methods that utilize mass array and/or next generation-based sequencing technology.

## Conclusion

To our knowledge, this is the first study to compare the epigenetic stability among ICRs between benign and malignant tumor cells driven by the same genomic mutation, *KrasG12D*. The results from the DNA methylation analyses of this study demonstrate that ICRs remain stable during the first reconstruction of the DNA methylation landscape but are targeted specifically by DNA hypermethylation during the second reconstruction of the DNA methylation landscape—a hallmark that defines the transition of benign tumor cells to malignant. Further investigation is required to determine whether the observed changes in DNA methylation at ICRs directly enhance the infiltrative potential of tumor cells. However, considering the effects that aberrant DNA methylation at an ICR can have on an entire imprinted domain, it is likely that concomitant hits to several ICRs can result in an aberrant protein environment within a tumor cell whereby several tumor suppressors are shut down and several oncogenes are activated. These dynamic changes in critical imprinted gene products may very well enhance a tumor cell’s ability to infiltrate tissue boundaries. Lastly, monitoring DNA methylation at ICRs may be a useful diagnostic in the clinical setting in determining the progression of a neoplasm.

## Methods

### Mouse strains and breeding

Two mouse strains were purchased from Jackson laboratories: B6.129-Krastm4Tyj/Nci (LSL- KrasG12D) and STOCK Tg(MMTV-Cre)4Mam/J (MMTV-Cre) [22, 23]. The LSL-KrasG12D mice were maintained as heterozygotes and bred with homozygous MMTV-Cre mice.

### Genotyping

Mice weaned at 21 days postpartum were separated by sex and marked by a hole punch with varying positions in the left ear. Ear clips were taken from the right ear for genomic DNA isolation and subsequent PCR analysis. Sample preparation and PCR protocols have been previously described [15]. Primer sets for genotyping can be found in Additional file 5: Table S2.

### Necropsy and histopathology

Mice were monitored daily for signs of distress or weight loss equaling 15% of total body weight and sacrificed according to the guidelines set forth in the IACUC protocol #16–060. Upon euthanasia, mice were submitted to full necropsy. Samples of squamous papilloma and thymic lesions were placed directly in tail lysis buffer with Proteinase K for subsequent DNA isolation. Detailed buffer compositions and DNA extraction protocols were previously reported [15]. The remaining portion of these lesions and representative samples of all other tissues were fixed for at least 48 h in 10% neutral buffered formalin (Thermo Scientific, Cat. # 5725) and then transferred to 70% ethanol. Tissues were further processed according to standard protocols for hematoxylin and eosin (H&E) staining and examined by a board-certified pathologist (IML).

### DNA methylation analysis

Combined Bisulfite Restriction Analysis (COBRA) and bisulfite sequencing was performed for DNA methylation analyses [28]. Genomic DNA from the thymic lymphoma and squamous papilloma lesions was isolated using a commercial kit (Genomic DNA clean and concentrator-25, Zymo Research, Cat. No. D4065), and subsequently, bisulfite-converted using another commercial kit (EZ DNA methylation kit, Zymo Research, Cat. No. D5002). Bisulfite-converted DNA was then used as template for PCR. Additional file 5: Table S2 contains detailed information pertaining to the genomic locations analyzed and their respective oligonucleotide bisulfite PCR primer sets. Each bisulfite PCR product was digested with at least one restriction enzyme. The resulting fragments were visualized using gel electrophoresis, and band densities were calculates as described below. Images were exported as tiff files into ImageJ software. Briefly, data was inverted, the background was removed, the brightness/contrast was adjusted, the bands were selected using the rectangular tool to generate density plots, density peaks were gated using the line tool, and the area of each peak was then calculated using the wand tool. The results were then copied into a Microsoft Excel spreadsheet for further processing and statistics. DNA methylation values were calculated in the following manner: 100*((area of peak from digested DNA/s)/(area of peak from digested band/s + area of peak from undigested DNA)). These % methylation values were then used for statistical analyses. First, a single factor ANOVA test was performed to identify significant differences among the sample. Upon a significant *P* value from ANOVA, the means of the tumor samples were then compared to the normal sample using the Student *t* test (two sample assuming equal variance). Three independent technical replicates were performed for each sample and each primer set. For the squamous papilloma panel, 15 samples were analyzed initially; however, only seven samples were used for statistical comparisons based on the consistent results from successful bisulfite PCRs across all primer sets (Additional file 6: Table S3). For bisulfite sequencing, PCR products were individually cloned using a commercial vector system (pGEM®-T Easy Vector System I, Promega, A1360). Ten clones were selected for sequencing. In brief, plasmids were purified using a commercial kit (DNA-spin plasmid DNA purification kit, iNtRON Biotechnology, 17097), restriction digest was performed to check for insert, BigDye Terminator v3.1 reactions were performed, products were precipitated and re-suspended in ABI Hi-Di formamide and sequences using a 3130XL Genetic Analyzer. Sequencing results were then manually processed to remove vector sequence, placed in the same orientation, and aligned using ClustalW. The methylation of each CpG site in the sequencing reads was then manually scored in an Excel spreadsheet. Sequencing reads with less than 95% bisulfite conversion efficiency were removed.

## Additional files

Additional file 1: Figure S1. DNA methylation signatures at ICRs in benign squamous papilloma and infiltrative thymic T cell lymphoma. Representative DNA methylation data from 11 ICRs in squamous papilloma and thymic lymphoma generated by COBRA. Data from 7 out of the 15 squamous papilloma tumors are shown and compared to a normal sample denoted with an N. Data from 3 representative thymic lymphoma samples are shown: A – hyperplastic, B – atypical hyperplastic, and C – neoplastic. The red C denotes where hypermethylation at ICRs occurred. Unmethylated DNA is denoted with a blue U, and methylated DNA is denoted with a red M. (PDF 26309 kb)

Additional file 2: Table S1. Mean percent methylation with 95% confidence intervals for each locus and sample. (XLSX 67 kb)

Additional file 3: Figure S2. Representative COBRA data from the *H19* – ECR1 and the *Zim3*/*Zfp264* – promoter. Squamous papilloma samples are numbered 1–15 and compared to a sample of normal skin (N). The numbers underneath the gel images indicate percent methylation for each sample. Red numbers indicate the samples that showed significant DNA methylation change based on *P* values less than 0.05. Unmethylated DNA is denoted with a blue U and methylated DNA is denoted by a red M. (PDF 5050 kb)

Additional file 4: Figure S3. Methylation profiles of ICRs, DMRs, promoters, and ECRs within three imprinted domains from squamous papilloma and thymic lymphoma tumors. Seven squamous papilloma tumors samples (labeled 1–7) from oral mucosa are compared to normal oral mucosa tissue (labeled N) from a wild-type littermate. Three thymic lymphoma tumor samples (A – hyperplastic, B – atypical hyperplastic, and C – neoplastic) are compared to normal thymic tissue (labeled N) isolated from a wild-type littermate. Mean percent methylation from three technical trials is plotted on the y-axis with 95% confidence intervals. Sample legends are presented at the bottom of the figure. (PDF 577 kb)

Additional file 5: Table S2. Genomic locations analyzed. This table contains the bisulfite PCR primer sets used along with the restriction enzyme used for each PCR product. (XLSX 17 kb)

Additional file 6: Table S3. This table contains the quantified COBRA data and statistical analyses for each locus analyzed. (XLSX 300 kb)
