# Supplementary material for: Epigenetic response of imprinted domains during carcinogenesis
Source: Clin Epigenetics. 2017 Aug 25;9:90. doi: 10.1186/s13148-017-0393-8 (PMC5572065; doi:10.1186/s13148-017-0393-8)
Supplement: Supplementary file 1 — DNA methylation signatures at ICRs in benign squamous papilloma and infiltrative thymic T cell lymphoma. Representative DNA methylation data from 11 ICRs in squamous papilloma and thymic lymphoma generated by COBRA. Data from 7 out of the 15 squamous papilloma tumors are shown and compared to a normal sample denoted with an N. Data from 3 representative thymic lymphoma samples are shown: A – hyperplastic, B – atypical hyperplastic, and C – neoplastic. The red C denotes where hypermethylation at ICRs occurred. Unmethylated DNA is denoted with a blue U, and methylated DNA is denoted with a red M. (PDF 26309 kb) [file 13148_2017_393_MOESM1_ESM.pdf]

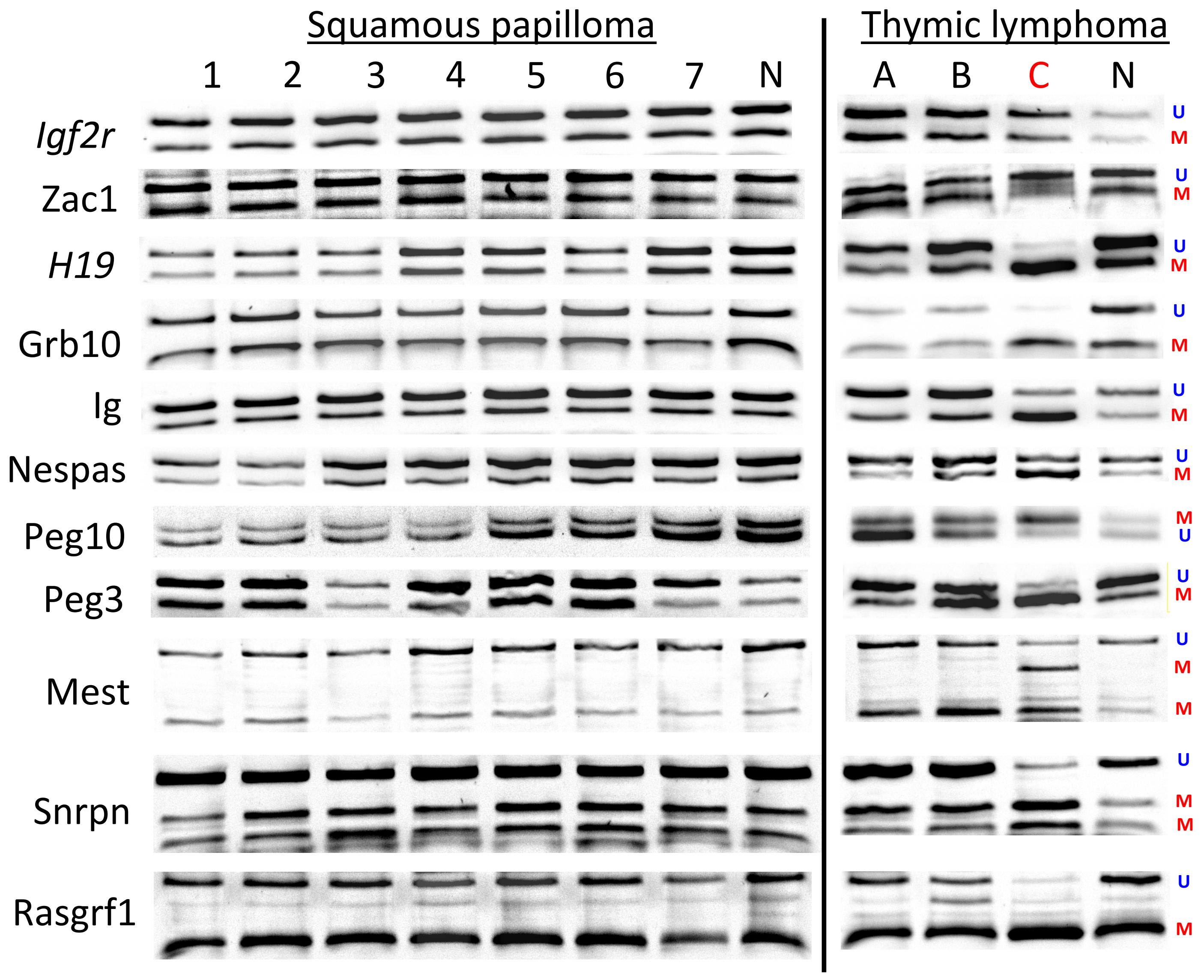

Squamous papilloma
1
2
3
4
5
6
7
N
Thymic lymphoma
A
B
C
N
Igf2r
Zac1
H19
Grb10
Ig
Nespas
Peg10
Peg3
Mest
Snrpn
Rasgrf1
U
M
U
M
U
M
U
M
U
M
U
M
M
U
U
M
U
M
M
U
M
M
U
M
