# Supplementary figures and images for: Epigenetic response of imprinted domains during carcinogenesis
Source: Clin Epigenetics. 2017 Aug 25;9:90. doi: 10.1186/s13148-017-0393-8 (PMC5572065; doi:10.1186/s13148-017-0393-8)

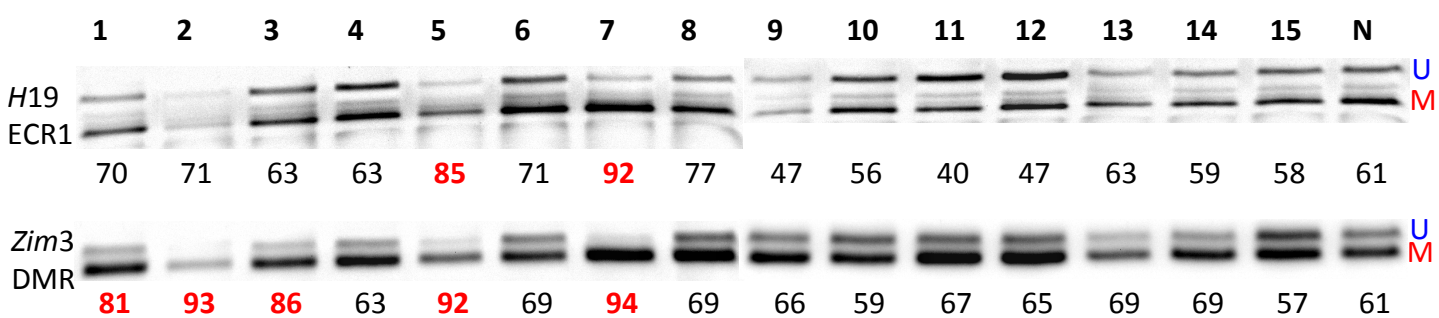

Supplement: Supplementary file 3 — Representative COBRA data from the H19 – ECR1 and the Zim3/Zfp264 – promoter. Squamous papilloma samples are numbered 1–15 and compared to a sample of normal skin (N). The numbers underneath the gel images indicate percent methylation for each sample. Red numbers indicate the samples that showed significant DNA methylation change based on P values less than 0.05. Unmethylated DNA is denoted with a blue U and methylated DNA is denoted by a red M. (PDF 5050 kb) [file 13148_2017_393_MOESM3_ESM.pdf]

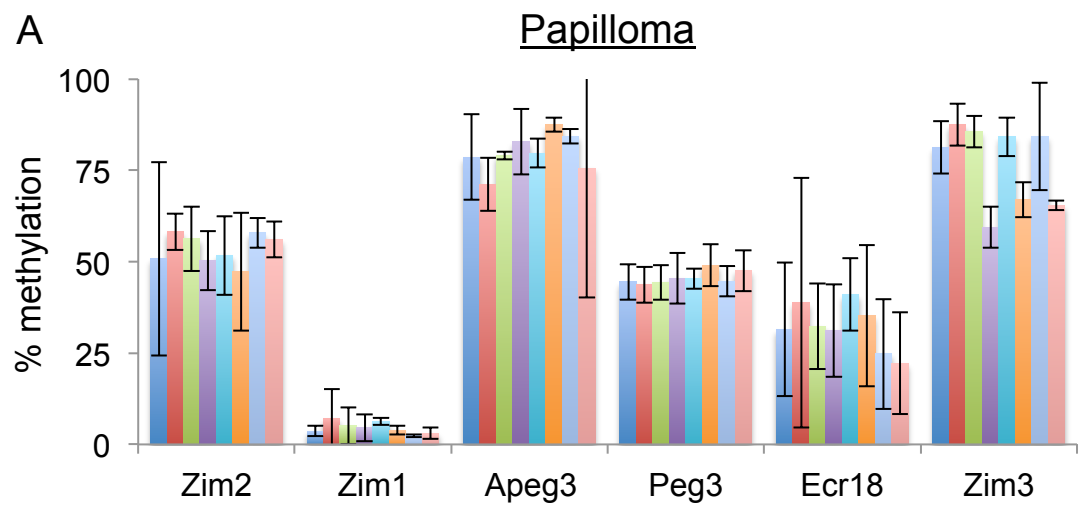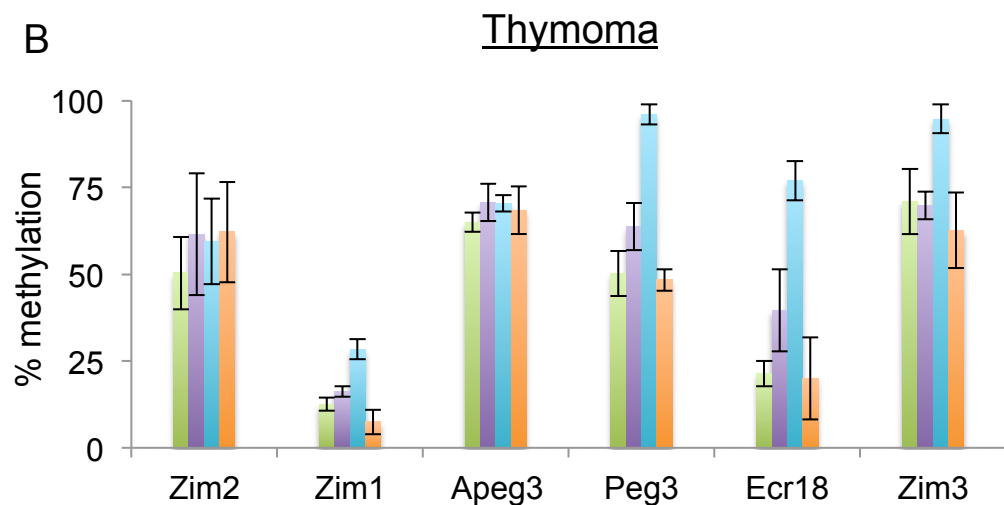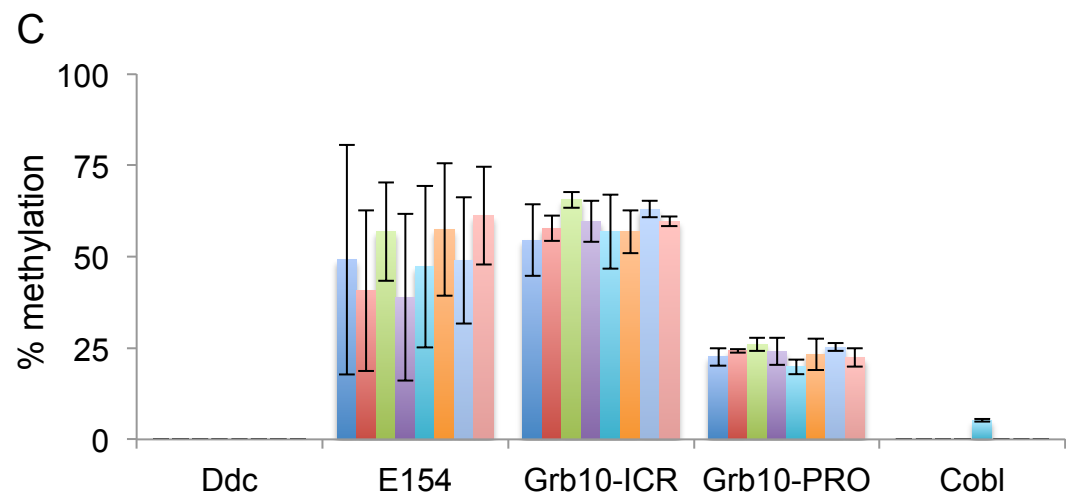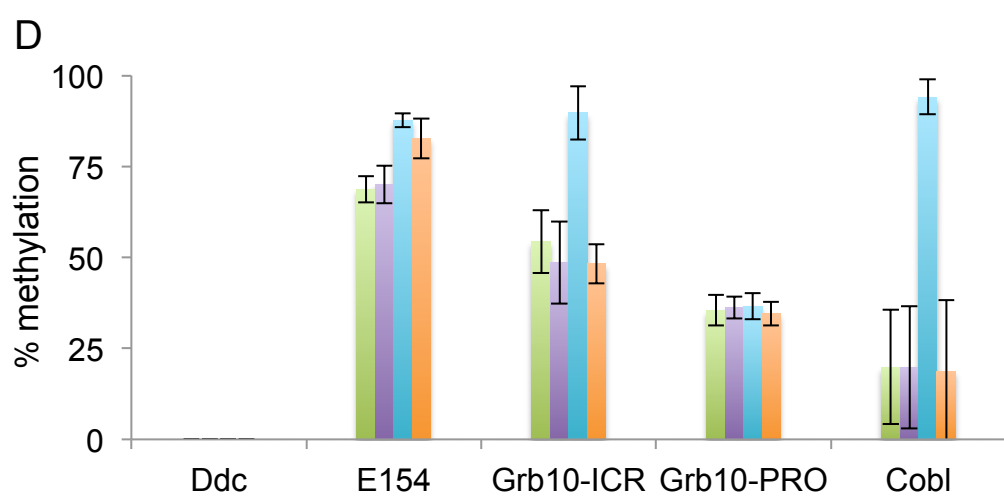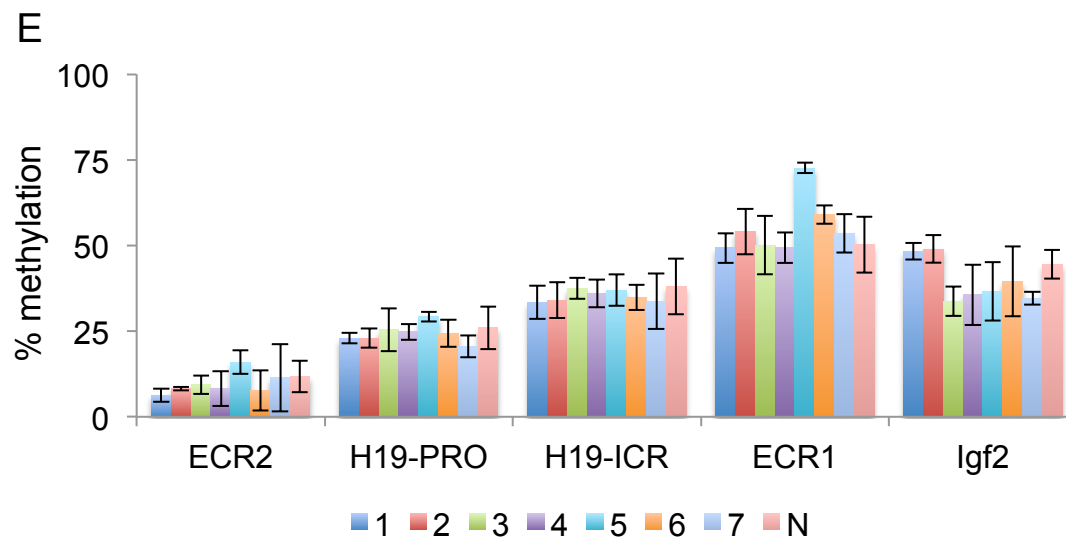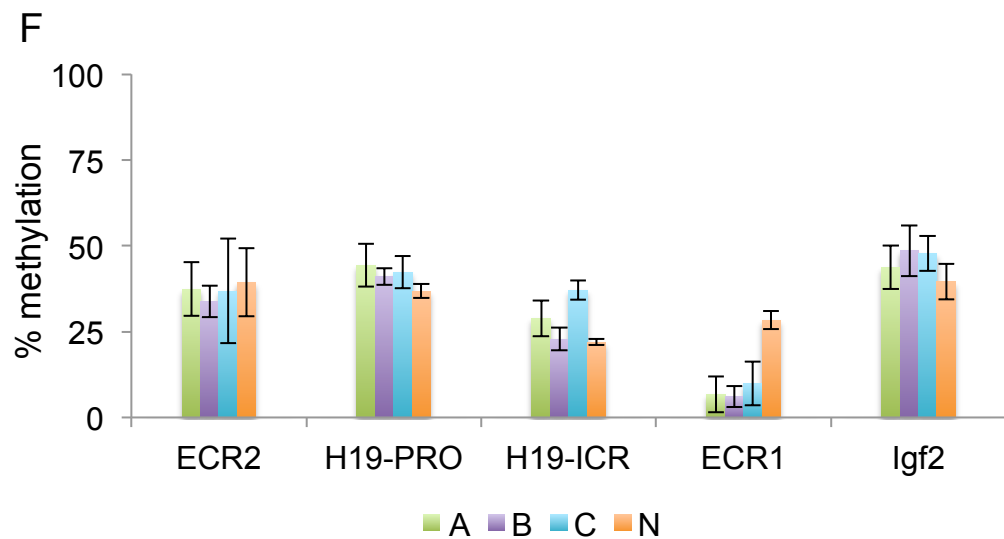

Supplement: Supplementary file 4 — Methylation profiles of ICRs, DMRs, promoters, and ECRs within three imprinted domains from squamous papilloma and thymic lymphoma tumors. Seven squamous papilloma tumors samples (labeled 1–7) from oral mucosa are compared to normal oral mucosa tissue (labeled N) from a wild-type littermate. Three thymic lymphoma tumor samples (A – hyperplastic, B – atypical hyperplastic, and C – neoplastic) are compared to normal thymic tissue (labeled N) isolated from a wild-type littermate. Mean percent methylation from three technical trials is plotted on the y-axis with 95% confidence intervals. Sample legends are presented at the bottom of the figure. (PDF 577 kb) [file 13148_2017_393_MOESM4_ESM.pdf]
